# Ex vivo microRNA and gene expression profiling of human Tr1‐like cells suggests a role for miR‐92a and ‐125a in the regulation of EOMES and IL‐10R

**DOI:** 10.1002/eji.202149315

**Published:** 2021-09-28

**Authors:** Marco De Simone, Michele Chirichella, Stefan Emming, Saveria Mazzara, Valeria Ranzani, Paola Gruarin, Giorgia Moschetti, Nadia Pulvirenti, Stefano Maglie, Chiara Vasco, Maria Cristina Crosti, Grazisa Rossetti, Massimiliano Pagani, Sergio Abrignani, Silvia Monticelli, Jens Geginat

**Affiliations:** ^1^ Istituto Nazionale Genetica Molecolare INGM ‘Romeo ed Enrica Invernizzi’ Milan Italy; ^2^ Institute for Research in Biomedicine (IRB) Università della Svizzera italiana (USI) Bellinzona Switzerland; ^3^ FIRC Institute of Molecular Oncology (IFOM) Milan Italy; ^4^ Department of Medical Biotechnology and Translational Medicine Università degli Studi Milano Italy; ^5^ Department of Clinical Sciences and Community Health Università degli Studi Milano Italy

**Keywords:** Tr1‐like cells, microRNAs, EOMES, IL‐10R

## Abstract

Ex vivo gene expression and miRNA profiling of Eomes^+^Tr1‐like cells suggested that they represent a differentiation stage that is intermediate between Th1‐cells and cytotoxic CD4^+^ T‐cells. Several microRNAs were downregulated in Eomes^+^Tr1‐like cells that might inhibit Tr1‐cell differentiation. In particular, miR‐92a targeted Eomes, while miR‐125a inhibited IFN‐g and IL‐10R expression.

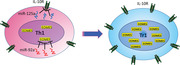

Regulatory T‐cells, comprising both FOXP3^+^Tregs and FOXP3^–^ type‐1 regulatory T‐cells (Tr1), are required to maintain immune homeostasis. We previously identified a population of human IL‐10 and IFN‐γ co‐producing Tr1‐like cells, which are involved in graft‐versus‐host disease, colitis, autoimmunity, and cancer [1, 2, 3, 4, 5]. They express the transcription factor eomesodermin (EOMES) [2, 6], which is characteristic for cytotoxic T‐lymphocytes (CTL) and controls IFN‐γ production and cytotoxic functions [7]. T‐bet expression, differentiation requirements, and clonotype sharing suggests that EOMES^+^ Tr1‐like cells are derived from Th1‐cells [2, 5, 6]. MicroRNAs (miRNAs) regulate gene expression and shape differentiation states, and are required for the functions of FOXP3^+^ Tregs [8]. The role of miRNAs in the biology of Tr1‐like cells is in contrast largely unknown.

Since different subsets of human CD4^+^T‐cells express EOMES [2], we asked how they were molecularly related. We purified EOMES‐expressing CD4^+^ T‐cell subsets, that is, Th1 effector memory cells (Th1_EM_), CD4^+^CTL, and Tr1‐like cells ex vivo from peripheral blood of healthy donors according to an established gating strategy [2] (Supporting Information Fig. 1A) and performed gene expression analysis. Th1 central memory cells (Th1_CM_), which largely lacked EOMES expression (Supporting Information Fig. 1B), were analyzed as control. We identified 424 differentially expressed genes (*p* < 0.01, Supporting Information Table 1). Hierarchical clustering revealed limited donor‐to‐donor variability (Fig. 1A), suggesting that the analyzed subsets represent conserved differentiation stages. This analysis resulted in one major cluster containing all EOMES‐expressing subsets, and a second cluster containing Eomes^–^Th1_CM_. In the EOMES^+^ subcluster, Tr1‐like cells clustered together with CTL. Notably, principal component analysis (PCA) positioned Th1_CM_ and CTLs at opposite sites of the three‐dimensional space, and Tr1‐like cells were positioned between Th1_EM_ and CTL (Fig. 1B), suggesting that they represent an intermediate differentiation state. Indeed, the majority of differentially expressed genes were downregulated in Tr1‐like cells as compared to Th1‐cells, but upregulated as compared to CTL (Supporting Information Fig. 1C and Table 2). Tr1‐like cells expressed higher levels of GZMK as compared to Th1_CM_ and CTL, and of IL‐10R as compared to Th1‐cells. Moreover, they expressed higher levels of *EOMES*, *GZMA*, *NKG7*, *CCL5*, and *HLA‐G* a*s* compared to Th1_CM_, but had downregulated *FOXO1* and *LTA*. CD4^+^CTL expressed the lowest levels of *CCR7*, *CD27*, and *LEF1*, suggesting that they are terminally differentiated effector cells. Selected differentially expressed genes and relevant controls were then measured by RT‐qPCR in independent donors (Supporting Information Fig. 2A). *GZMK* and *EOMES* were highly expressed in Tr1‐like cells, as expected [2]. *IFNG* mRNA was constitutively expressed in CTL and Tr1‐like cells, whereas *IL10* and *GZMB* mRNA were largely restricted to Tr1‐like cells and CD4^+^CTL, respectively. miRNA expression in human CD4^+^ T‐cell subsets is superior compared to gene expression patterns to map CD4^+^ T‐cell differentiation stages [9]. We therefore analyzed the expression of 664 miRNAs in the same T‐cell subsets. Twelve miRNAs were found to be differentially expressed, as detected by TaqMan miRNA arrays (Fig. 1C **;** Supporting Information Table 3). Hierarchical clustering revealed again that Tr1‐like cells clustered together with CD4^+^CTLs. Most of the differentially expressed miRNAs were downregulated in Tr1‐like cells and in CTL. Three of these miRNAs were highly expressed in Th1_CM_, suggesting that they might be involved in repressing cytotoxic cell fates. Conversely, miR‐186, miR‐194, and miR‐345 were highly expressed, although not uniquely, in Tr1‐like cells. Validation of selected miRNAs by RT‐qPCR in independent donors confirmed downregulation of miR‐150, miR‐31, and, most notably, miR‐92a and miR‐125a in Tr1‐like cells (Fig. 1D). Inspection of the putative targets using TargetScan revealed that both miR‐125a and miR‐92a targeted Tr1‐expressed genes. Specifically, the intersection of differentially expressed genes with the top 500 TargetScan predicted targets (irrespective of site conservation) of the miR‐125 family and of miR‐92a‐3p identified genes involved in Tr1‐like cell biology. Thus, putative targets of the miR‐125 family included *IL10RA*, while a putative target of miR‐92a was *EOMES* (Fig. 2A). The 3’‐untranslated region (3’UTR) of the *EOMES* mRNA contains a putative miR‐92a responsive element (Supporting Information Fig. 2B). We therefore performed dual luciferase assay to assess whether this region was a target of miR‐92a. Upon transfection in HEK‐293T‐cells, a synthetic miR‐92a mimic oligonucleotide significantly reduced luciferase expression from a reporter plasmid containing the 3’UTR of the human *EOMES* gene, as compared to a scrambled control oligonucleotide (Fig. 2B). To investigate whether miR‐92a could affect EOMES protein expression in primary human T‐lymphocytes, we isolated CCR5^+^CD4^+^T‐cells, which are enriched for Eomes^+^ cells (Supporting Information Fig. 2C). After transfection with either a miR‐92a mimic or scrambled control oligonucleotide, the levels of EOMES protein expression were moderately, but consistently, reduced (Supporting Information Fig. 2D), suggesting that this miRNA could indeed suppress EOMES expression in CD4^+^T‐cells. Next, we focused on miR‐125a. Notably, its closely related family member miR‐125b is expressed exclusively in naïve CD4^+^T‐cells [9] (Fig. 2C). Conversely, miR‐125a was also expressed in Th1‐cells, but remained low in CTL and Tr1‐like cells. The seed sequences (nucleotide 2‐to‐7 of the miRNAs, responsible for target specificity) of miR‐125a and miR‐125b are identical, as expected for a miRNA family, suggesting that they possess similar target specificities. Therefore, potential differences in their mRNA targeting are rather due to their different expression patterns. The IL‐10R is highly expressed on regulatory T‐cells, including Tr1‐like cells (Fig. 2D **;** Supporting Information Fig. 2E), and it is required to maintain IL‐10 production and suppressive capabilities [10]. Moreover, the *IL10RA* gene was shown to be targeted by miR‐125b in human CD4^+^ T‐cells [9]. To assess the ability of miR‐125a to regulate the expression of *IL10RA*, we performed luciferase reporter assay using a plasmid containing the 3’UTR of this gene, either wild‐type or mutated in the region complementary to the miR‐125 seed sequence [9]. Co‐transfection of miR‐125a strongly and significantly reduced reporter expression from the wild‐type, but not from the mutated, 3’UTR (Fig. 2E). To assess the role of miR‐125a in primary human T‐cells, we transfected CD4^+^CD45RA^–^ memory T‐cells with either a miR‐125a mimic, an antagomir to inhibit miR‐125a activity or with scrambled controls. After 2 days, the expression of miR‐125a was strongly elevated in miR‐125‐mimic transfected cells, and diminished upon antagomir transfection (Supporting Information Fig. 2F). Under these conditions we monitored the expression of the predicted targets by RT‐qPCR and by flow cytometry. Both IFN‐γ and IL‐10Rα were slightly reduced both at the mRNA and protein level upon transfection with the miR‐125a‐mimic, and were instead slightly elevated with the miR‐125a antagomir (Supporting Information Fig. 2G). In conclusion, by performing gene expression and miRNA profiling of *ex vivo* isolated human EOMES^+^Tr1‐like cells, we provide additional evidence that Tr1‐like cells are a unique T‐cell subset. Moreover, our data suggests that miR‐92a and miR‐125a target the expression of Tr1‐associated genes like *EOMES* and *IL‐10R*, and might thus act as inhibitors of Tr1 differentiation.

**Figure 1:**
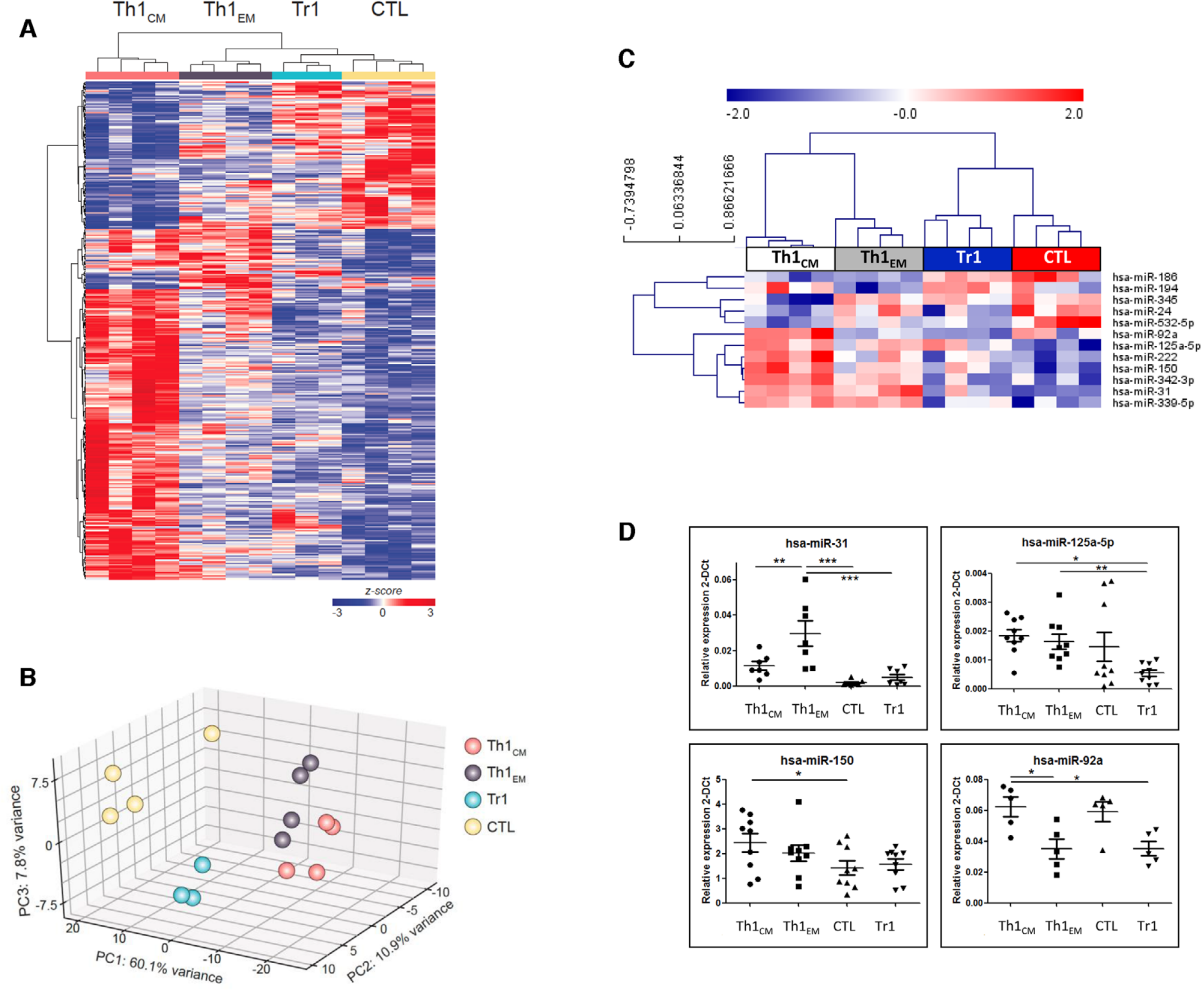
**Gene expression and miRNome analysis of human EOMES^+^CD4^+^ T‐cell subsets. (A)** Hierarchical clustering of differentially expressed genes in EOMES^+^ Tr1‐like cells (*n* = 3), CD4^+^CTL, Th1_CM_, and Th1_EM_ (*n* = 4) according to one‐way ANOVA (*p* < 0.01). (**B)** Three‐dimensional PCA of selectively expressed genes. (**C)** Hierarchical clustering of 12 miRNAs expressed in Th1_CM_, Th1_EM_, CD4^+^CTL, and Tr1‐like subsets, selected by one‐way ANOVA (*p* < 0.01). Data, normalized on global mean, are presented as *z*‐scores calculated on ΔCt. (**D)** Differential expression of four selected miRNAs (miR‐31 (*n* = 7), miR‐125a‐5p (*n* = 8), miR‐150 (*n* = 9), and miR‐92a (*n* = 5)) in independent donors were analyzed by RT‐qPCR (data represented as 2^–ΔCt^). Statistical analysis was performed using a one‐way ANOVA and Tukey post‐test between four groups: Th1_CM_, Th1_EM_, CTL, and Tr1 (*p < 0.05; **p < 0.01; ***p < 0.001).

**Figure 2:**
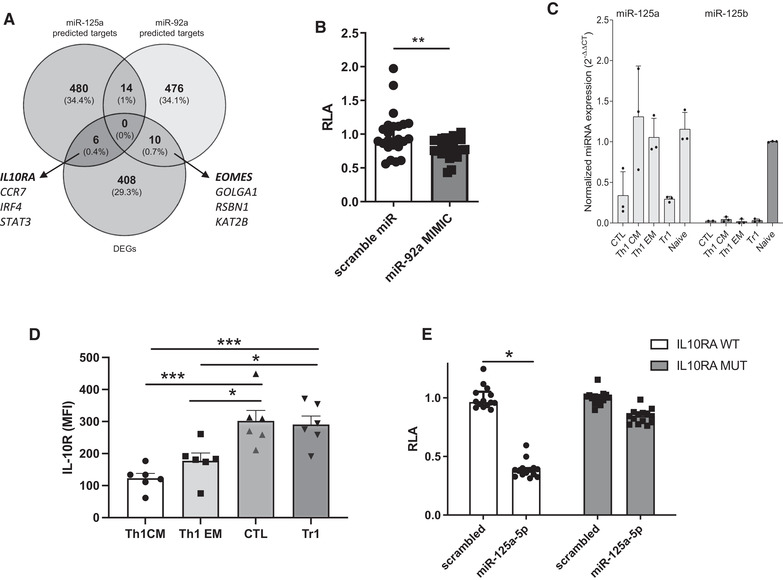
**Identification of putative gene targets of miR‐92a and miR125a. (A)** Venn Diagram showing the overlap between differentially expressed genes and the miRNA targets predicted by TargetScan. (**B)** Dual‐luciferase assay in HEK‐293T cells transfected with the human *EOMES* 3’UTR together with miR‐92a or a scrambled control. Mean of three independent experiments with six to nine technical replicates. Statistical analysis was performed using a Wilcoxon matched‐pairs signed rank test (*p < 0.05). Error bars show median and interquartile range. (**C)** Expression of miR‐125a and miR‐125b in the indicated CD4^+^ T‐cell subsets was measured by RT‐qPCR (3 independent donors analyzed in 3 experiments). **(D)** IL‐10Rα protein levels in gated CD4^+^CTL, Tr1‐, Th1_EM_, and Th1_CM_‐cells and measured by flow cytometry (*n* = 6, 1 experiment). Shown is the MFI; Fluorescence minus one was used as negative control. the statistical analysis was performed using a one‐way ANOVA. (**E)** Dual‐luciferase assay in HEK‐293T cells transfected with the human *IL‐10RA* 3’UTR together with a miR‐125a or scrambled control. Data show four independent experiments with three to four technical replicates. Error bars show median and interquartile range. Statistical analysis was performed using a Kruskal‐Wallis test (**p < 0.005).

## Conflict of interest

The authors declare no commercial or financial conflict of interest.

### Peer review

The peer review history for this article is available at https://publons.com/publon/10.1002/eji.202149315

**Abbreviations**
3’‐UTR: 3’‐untranslated region
CTL: cytotoxic T‐lymphocytes
EOMES: eomesodermin
miRNA: microRNA
Tr1: FOXP3^–^ type‐1 regulatory T‐cells

## Supporting information

Supporting Information

Supporting Information

Supporting Information

Supporting Information

Supporting Information
