# Supplementary material for: Ex vivo microRNA and gene expression profiling of human Tr1‐like cells suggests a role for miR‐92a and ‐125a in the regulation of EOMES and IL‐10R
Source: Eur J Immunol. 2021 Sep 28;51(12):3243–6. doi: 10.1002/eji.202149315 (PMC9293355; doi:10.1002/eji.202149315)
Supplement: Supplementary file 1 — Supporting Information [file EJI-51-3243-s004.pdf]

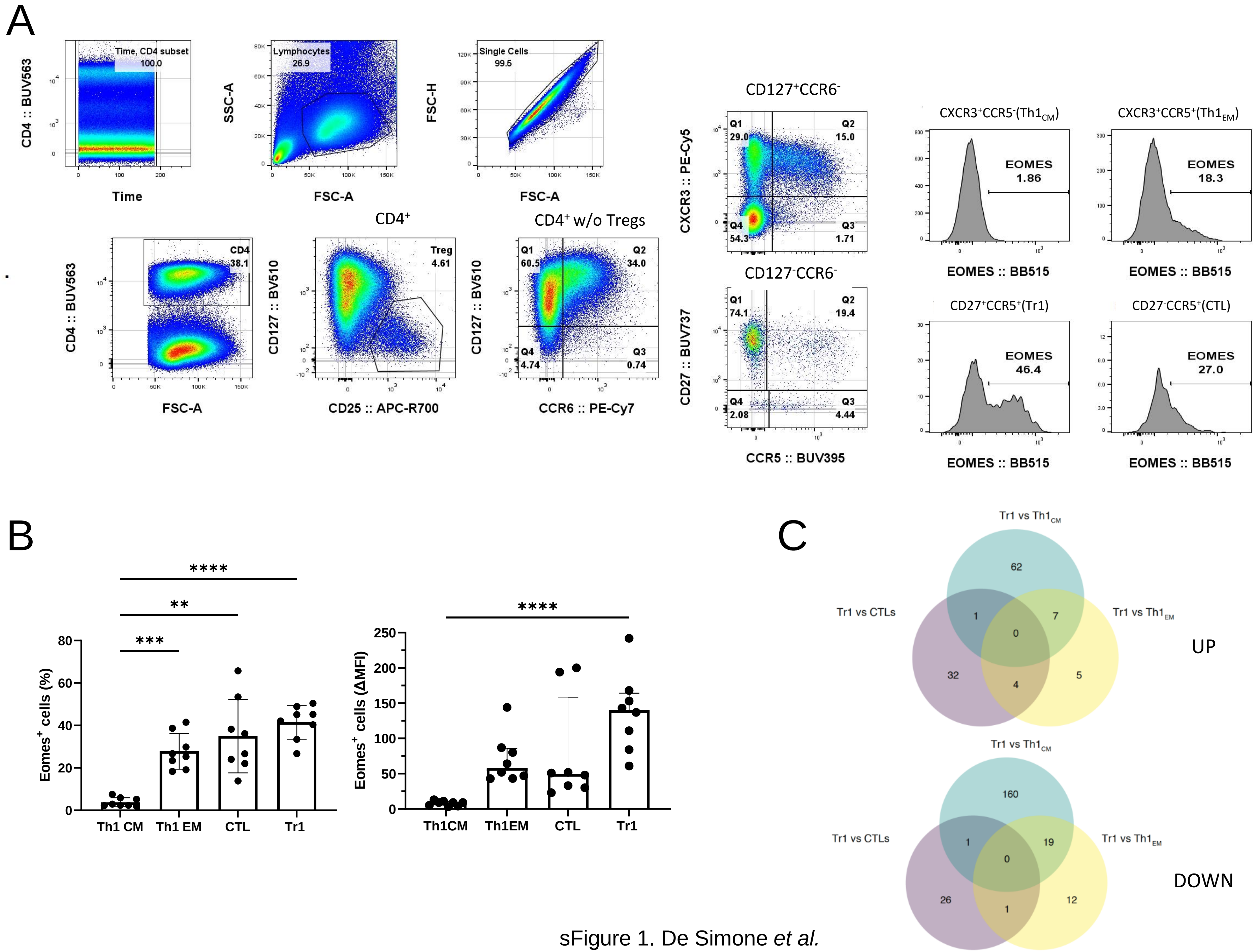

sFigure 1. De Simone *et al.*

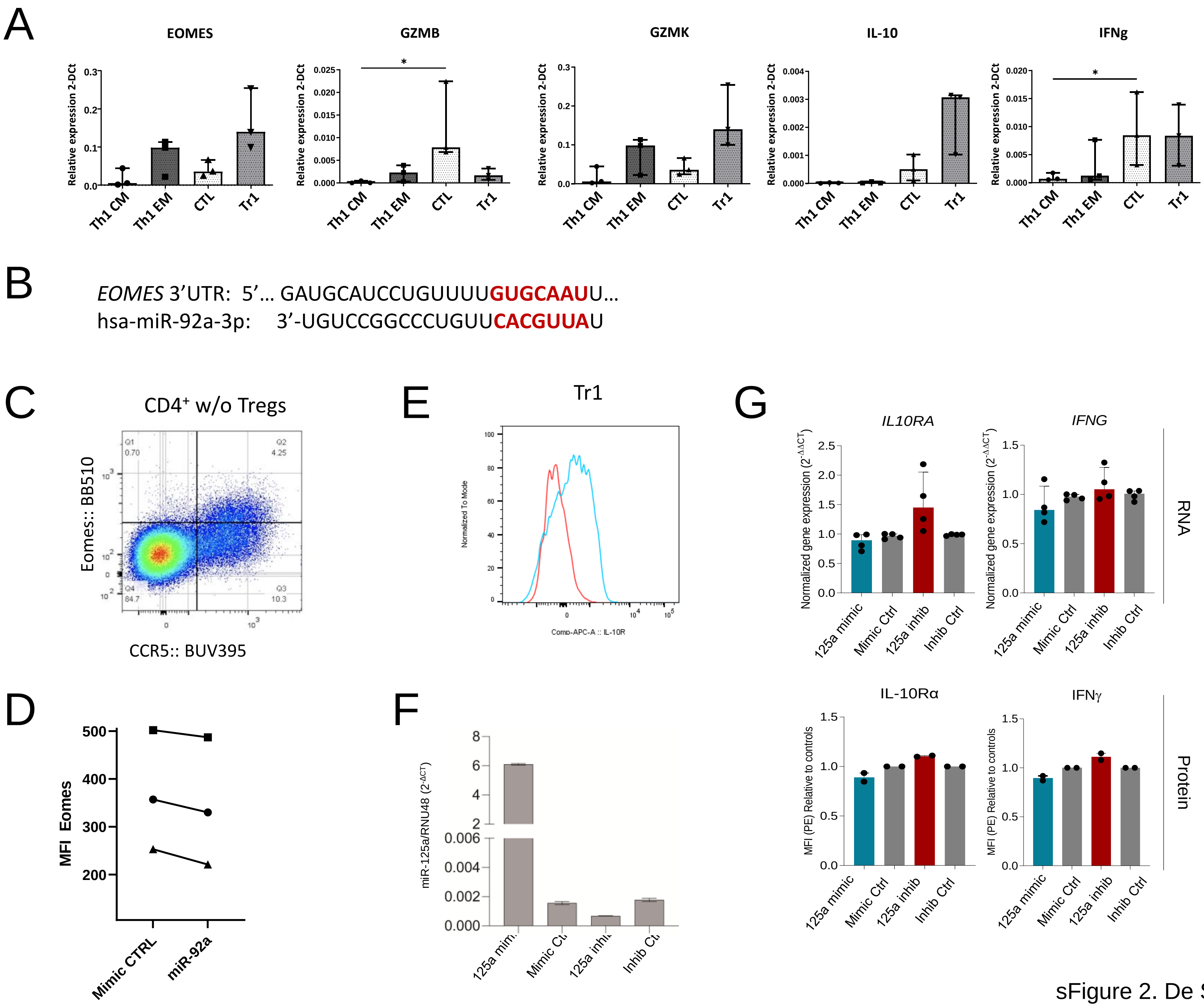

sFigure 2. De Simone *et al.*

Supporting Information

sFigure 1: Gating strategy, EOMES expression and differentially expressed genes of the 4 analyzed CD4$^+$T-cell subsets A: Gating strategy **for** CD4$^+$CTL, Tr1-, Th1$_{EM}$, and Th1$_{CM}$-cells **B.** *Ex vivo* EOMES protein levels in gated CD4$^+$CTL, Tr1-, Th1$_{EM}$, and Th1$_{CM}$-cells from peripheral blood (n=8, 2 experiments). Shown are the percentage of Eomes$^+$ cells (left) and the MFI (right); statistical analysis was done by One-way ANOVA (%, *left*) or with a Friedman Test (MFI, *right*). **C.** Venn Diagram showing the overlap between the differentially expressed up- and down-regulated genes in Tr1-cells, CTL, Th1$_{EM}$ and Th1$_{CM}$-cells.

sFigure 2: Validation of gene expression and miRNA targeting in CD4$^+$ T-cells A. Validation of selected expressed and control genes (*EOMES*, *GZMK*, *GZMB*, *IL10*, *IFNG*) by RT-qPCR in independent donors (n=4), statistical analysis was done by with a Friedman Test. **B.** Alignment of miR-92a with the human *EOMES* 3'UTR. The miRNA seed sequence and its target sequence are indicated in red. **C.** Representative *ex vivo* Eomes expression in conventional (CD4 w/o Tregs, see sFig 1A)) CCR5$^+$ T-cells **D.** Eomes protein expression levels in purified human CD4$^+$CCR5$^+$ T-cells that were transfected with miR-92a or a scrambled control. Data shows 3 different donors analysed in 3 independent experiments. **E.** Histogram Overlay showing *ex vivo* IL-10R expression in gated Tr1-cells. The red line shows the negative Control (Fluorescence minus One), whereas the blue line shows IL-10R staining. **F.** Memory T-cells (CD4$^+$CD45RA$^-$) were transfected with a miR-125a mimic or antagomir oligonucleotide, or controls. 48 h after transfection, the expression of miR-125a was measured by RT-qPCR. 4 different Donors analysed in 2 independent experiments. Error bars report standard deviations. **G.** Same as D., except that expression of IL-10R and IFN-g were measured both at mRNA (top) and protein (bottom) level. Error bars report standard deviations.

sTable 1: List of 424 differentially expressed genes Differentially expressed genes in Tr1-like cells were identified by One-way ANNOVA (p<0.01) followed by a post-hoc analysis (Tukey HSD) in order to compare every mean to every other mean. Genes of interest are highlighted in yellow.

sTable 2: Differentially expressed genes in Tr1-cells compared to CTL, Th1$_{EM}$ or Th1CM Differentially expressed genes in Tr1-like cells identified by One-way ANNOVA (p<0.01) as compared to the individual subsets (CTL, Th1$_{EM}$ or Th1CM). Genes identified in two subsets are highlighted with the indicated colours.

sTable 3: Differentially expressed miRNAs

sTable 4: List of all analysed genes

Materials and Methods

T-cell subsets isolation

Buffy-coat blood of healthy donors was obtained from the Ospedale Maggiore in Milan (Italy) or from the Swiss Blood Donation Center of Basel and Lugano (Switzerland), with informed consent

from the Swiss Red Cross and authorization number CE 3428 from the Comitato Etico Canton Ticino. Peripheral blood mononuclear cells were isolated by Ficoll-hypaque density gradient centrifugation. Human blood primary lymphocyte subsets were purified to >95% by cell sorting using a previously published combination of surface markers [2] according the Guidelines of Eur. J. Immunol. [11]. Thus, Th1-cells were purified as $CD4^{+}IL7R^{+}CD25^{-}CCR6^{-}CXCR3^{+}$ cells and subdivided into $Th1_{CM}$ and $Th1_{EM}$ cell subsets according to CCR5 expression [12]. $CD4^{+}IL7R^{-}CD25^{-}CCR6^{-}CCR5^{+}$ T-cells were subdivided into CTL and Tr1-like cells according to CD27 expression [2]. Antibodies are further listed: anti-human CD4 OP, clone OKT4 (Biolegend); anti-human CD25 FITC, clone BC96 (Biolegend); anti-human CD127 biotin, clone HIL-7R-M21 (BD Pharmingen); streptavidin-Allophycocyanin (APC), (Biolegend); anti-Human CD195 (CCR5) PE-Cy7, clone 2D7/CCR5 (BD Pharmingen); anti-Human CD196 (CCR6) PE, clone R6H1 (eBioscience) and anti-human CD183 (CXCR3) PE-Cy5, clone 1C6/CXCR3 (BD Pharmingen).

RNA isolation and miRNA-mRNA expression profiling

Total RNA was isolated using mirVana miRNA Isolation Kit (Ambion) following standard protocols. Briefly, the cell lysates were extracted once with acid phenol-chloroform and further purified to yield total RNA with specific miRNA retention. Extracted RNA was quantified with RiboGreen Quantitation Kit (Molecular Probes) on an Infinite F200 plate reader (Tecan Trading AG). All extracted RNA samples were quality controlled for integrity with a 2100 Bioanalyzer (Agilent Technologies) and samples with RIN (RNA Integrity Number) lower than 8 were discarded. The standard Megaplex protocol (Applied Biosystems) was performed starting from 10 ng of total RNA for each sample with pre-amplification.

Gene expression of whole transcriptome was performed using Illumina Direct Hybridization Assays according to the standard protocol (Illumina Inc.). Total RNA was isolated, quality controlled and quantified; for each sample 80 ng of total RNA were reverse transcribed according to the Illumina TotalPrep RNA Amplification kit (Ambion) and cRNA was generated after 14 h of *in vitro* transcription. Washing, staining and hybridization were performed according to the standard Illumina protocol: briefly, 750 ng of cRNA of each sample in a final volume of 15 µl were hybridized onto Illumina HumanHT-12 v3 Expression BeadChip arrays. Hybridization and scanning were performed according to the manufacturer's indications on an Illumina iScan System and data were processed with BeadStudio v.3.

For miRNA analysis, total RNA was reversed transcribed using the Megaplex RT stem-loop primers in a 7.5 µl reaction volume through the protocol's default 40 cycles run; 2.5 µl of each RT product were pre-amplified in a 25 µl reaction volume with Megaplex PreAmp primers to increase detection sensitivity according to manufacturer's specifications. Expression analysis of mature miRNAs was assessed using TaqMan Low Density Arrays (Applied Biosystems), enabling specific detection of 664 human miRNAs (miRBase v.10.1) and 3 small RNA controls common to all plates (RNA44, RNA48, MammU6). 9 µl of 4-fold diluted pre-amplified RT product were 100-fold diluted in the PCR reaction mix and amplified using TaqMan Low Density Arrays (TLDAs) on a 7900HT (Applied Biosystems) according to the standard protocol.

Data filtering and statistical analysis

Gene expression profiling. Gene expression arrays were quantile normalized with background subtraction, and average signals were calculated on gene-level data for genes whose detection p-value was lower than 0.001 in at least one of the cohorts considered ($Th1_{CM}$, $Th1_{EM}$, CTL and Tr1-like cells). Normalized data were log2 transformed. A one-way ANOVA (p-value< 0.01) was used to select for all four cell subsets profiled. Unsupervised hierarchical clusters on significant genes were performed with euclidean distance and ward.D2 linkage. In order to survey possible groupings in the data, principal component analysis (PCA) was performed. Statistical test, hierarchical clustering and PCA were carried out using R 4.0.3 software.

miRNA profiling. Raw CT values were calculated using the SDS software v.2 and exported as "Amplification Data" text files for each plate. TLDAs affected by globally poor or anomalous amplification were discarded. After this quality control step, 16 samples belonging to the four distinct cell subsets entered the final dataset. A one-way ANOVA (p-value< 0.001) was used to select miRNA classifiers for all four cell subsets profiled. In order to minimize the biological variability for the classifiers selection we considered miRNAs expressed in at least 2/3 (66%) of the profiled samples analyzed in each cell subset. Unsupervised hierarchical clusters on significant miRNAs were performed with Pearson distance and complete linkage. Statistical test and hierarchical clustering were performed on MeV software version 4.5.

miRNA and gene expression RT-qPCR

For assessment of *IL10*, *IFNG*, *IL10RA*, *GZMK*, *GZMB*, *EOMES* and *18S* rRNA (used as normalizer) gene expression levels, TaqMan Gene Expression assays (Applied Biosystems) were used. 200 ng of total RNA were used for reverse transcription with VILO Reverse Transcriptase (Invitrogen). Diluted cDNA was then used as input for RT-qPCR. In some cases, expression of *IL10RA* and *IFNG* mRNAs was measured using the PerfeCTa SYBR Green FastMix (Quanta Bioscience) and the following primers: *IL10RA* FW: 5'-TTCAGCCTCCTAACCTCTGG; RV: 5'-TGGTCACGGTGAAATACTGC; *IFNG* FW: 5'-CGAGATGACTTCGAAAAGCTG; RV: 5'-CAGTTCAGCCATCACTTGGA.

For assessment of miR-31, miR-125a-5p, miR-125b-5p, miR-92a and RNU48 (used as normalizer) levels on specific human lymphocyte subsets single Taqman MicroRNA assays were used (Applied Biosystems). 5 ng of total RNA were used for reverse transcription with specific primers for miR-31, miR-125a-5p, miR-92a and RNU48 and Taqman MicroRNA Reverse Transcription Kit followed by real-time PCR reaction on a 7900HT Real Time PCR System according to the manufacturer's standard protocol.

T-cell transfection and analysis

Resting $CD4^+$ T lymphocytes ($3x10^5$ – $1x10^6$) either total memory (for miR-125a transfection) or $CCR5^+$ (for miR-92a) cells were transfected with 2 µM of miRNA mimic oligonucleotide, antagomiR or negative control #4 (Qiagen) using the Neon transfection system with the 10 µL kit and the

following settings: 2200 V, 20 ms, 1p. For transfections with miR-125a, T cells were cultured for two days in complete medium (RPMI-1640 supplemented with 5% human serum, 1% nonessential aminoacids, 1% sodium pyruvate, 1% glutamine, penicillin, streptomycin, 50 μM of β-mercaptoethanol) before analysis of protein expression by flow-cytometry and RNA extraction. For miR-92a, 24 h after transfection, T cells were activated for 4 h with plate-bound anti-CD3 (clone TR66, produces in-house) and anti-CD28 (1 μg/ml) antibodies, followed by intracellular staining using an anti-EOMES antibody (clone WD 19128, Ebioscience) using an intracellular staining kit for transcription factors (Invitrogen). For intracellular cytokine staining, cells were treated for 2.5 h with phorbol 12-myristate 13-acetate (PMA, 200 nM) and ionomycin (1 μg/ml), followed by addition of with brefeldin A (10 μg/mL) for another 2.5 h. Subsequently, cells were fixed in 4% paraformaldehyde for 10 min and permeabilized with 0.5% BSA and saponin. Antibody staining was performed for 20 min at room temperature.

Dual-luciferase reporter assay

The 3'UTR of the IL-10Rα [9] or *EOMES* genes were cloned in the psiCHECK-2 vector immediately downstream of the gene encoding the renilla luciferase. HEK-293T cells were transfected with 10 ng of the psiCHECK-2 construct along with 20 nM miR-92a or miR-125a mimic or scrambled control oligonucleotide (Qiagen) using Lipofectamine2000 (Invitrogen). After 24 h, cells were lysed and firefly and renilla luciferase activity was measured using the Dual-Luciferase Reporter Assay System (Promega), following exactly manufacture's instructions. Results are presented as the ratio between the renilla and firefly luciferase activities.

11. Cossarizza, A., et al., *Guidelines for the use of flow cytometry and cell sorting in immunological studies (second edition).* Eur J Immunol, 2019. **49**(10): p. 1457-1973.
12. Paroni, M., et al., *Recognition of viral and self-antigens by TH1 and TH1/TH17 central memory cells in patients with multiple sclerosis reveals distinct roles in immune surveillance and relapses.* J Allergy Clin Immunol, 2017.
